# Occurrence of amyotrophic lateral sclerosis during TNF inhibitor treatment in inflammatory rheumatic disease. What are the relationships?

**DOI:** 10.1016/j.ero.2025.11.019

**Published:** 2025-12-10

**Authors:** Eric Toussirot, Laurent Tatu, Matthieu Bereau

**Affiliations:** 1 INSERM Centre Investigation Clinique CIC-1431, Université Marie et Louis Pasteur, CHU de Besançon, Besançon, France; 2 Neurologie-électrophysiologie Clinique, Université Marie et Louis Pasteur, CHU de Besançon, Besançon, France

## Abstract

Neurological adverse events have been reported in patients receiving tumor necrosis factor inhibitors (TNFi) for the treatment of inflammatory rheumatic diseases. The occurrence of amyotrophic lateral sclerosis (ALS) during TNFi therapy is rare but raises the question of a possible relationship. We report 2 cases of ALS diagnosed during TNFi treatment: the first in a patient with spondyloarthritis treated with adalimumab and the second in a patient with seronegative polyarthritis treated with infliximab. Tumor necrosis factor alpha (TNFα) has been implicated in ALS pathogenesis and is considered to exert both neuroprotective and neurotoxic effects, depending on the differential expression of its receptors in distinct regions of the central nervous system. We also review data from pharmacovigilance databases and discuss the potential influence of TNFα inhibition on ALS development.

## INTRODUCTION

Tumor necrosis factor inhibitors (TNFi) are widely used in the treatment of various immune-mediated diseases, including rheumatoid arthritis (RA), spondyloarthritis (SpA), psoriasis, and inflammatory bowel diseases. Infections are the most common adverse effects associated with TNFi, particularly tuberculosis, but neurological events have also been reported, including demyelinating diseases, mononeuritis, and Guillain-Barré syndrome [1]. The occurrence of amyotrophic lateral sclerosis (ALS) during TNFi exposure is rare [[2], [3], [4]]. We report 2 new cases and discuss the potential role of tumor necrosis factor alpha (TNFα) in ALS, as well as whether TNFi could act as a predisposing or triggering factor for this neurodegenerative disorder.

### Case 1

A 49-year-old Caucasian woman had a history of arthritis affecting the elbows and knees. She had been diagnosed with ulcerative colitis more than 10 years earlier. Human leucocyte antigen-B27 was negative, but synovial fluid analysis of the knee revealed an inflammatory effusion. She was therefore diagnosed with peripheral SpA. Treatment with methotrexate and systemic glucocorticoids provided no improvement. Adalimumab 40 mg every other week was then initiated, resulting in the resolution of joint symptoms. After 3 years of treatment, she developed weakness in both lower limbs. Deep tendon reflexes were brisk, whereas touch, proprioception, and vibration sense were preserved. Fasciculations were observed in the upper limbs and tongue. Electrophysiological testing demonstrated pure axonal motor neuron dysfunction in all 4 limbs and the tongue. Spinal and brain magnetic resonance imaging, as well as biological, infectious, and immunological tests, were unremarkable. A diagnosis of ALS was made, riluzole was initiated, and adalimumab was discontinued. The disease progressed rapidly with dysphagia and generalized weakness, and the patient died 2 years after diagnosis (Table 1).

**Table 1:** Cases of development of amyotrophic lateral sclerosis in patients with inflammatory rheumatic disease treated by a TNF inhibitor

Case	1	2
Sex/age (y)	F/49	M/ 72
Inflammatory rheumatic disease	Peripheral SpA	Seronegative RA
TNFi	Adalimumab	Infliximab
Total TNFi exposure (months)	50	33
csDMARDs	No	MTX
Concomitant drugs	No	No
Timeframe between first symptom and start of TNFi (mo)	43	18
Genetic markers: *SOD1* and *C9ORF72* mutations	Negative	Negative
TNFi withdrawal	Yes	No
Type of ALS	Spinal and bulbar	Spinal and bulbar
Riluzole treatment	Yes	Yes
Time to death (mo)	24 mo	Still alive

ALS, amyotrophic lateral sclerosis; csDMARDs, conventional synthetic disease modifying antirheumatic drugs; M, male; F, female; MTX, methotrexate; RA, rheumatoid arthritis; SpA, spondyloarthritis; TNF, tumor necrosis factor; TNFi, tumor necrosis factor inhibitor.

### Case 2

A 72-year-old Caucasian man with no medical history was referred to the rheumatology department for symmetrical polyarthritis involving the hands, wrists, shoulders, knees, ankles, and feet, with morning stiffness. Physical examination revealed synovitis mainly affecting the hands and wrists. Rheumatoid factors and anticitrullinated protein antibodies were negative, leading to a diagnosis of seronegative polyarthritis. No erosions were seen on hand and foot X-rays. Methotrexate was initiated at up to 20 mg/wk subcutaneously, but disease activity persisted (Disease activity score 28 -Erythrocyte sedimentaton rate 3.2). Subcutaneous infliximab 120 mg every other week was introduced, resulting in significant improvement. Eighteen months later, he developed lower limb weakness with motor deficits of the anterior tibial muscles, brisk reflexes, and an extensor plantar response. Diffuse fasciculations were observed in the limbs. Electromyography showed widespread denervation in the arms and legs, consistent with pure axonal motor neuron dysfunction. Brain and spine magnetic resonance imaging were normal, as were infectious, immunological, and cerebrospinal fluid studies. A diagnosis of ALS was made, and riluzole was initiated. Infliximab was maintained. Fourteen months after the diagnosis, the patient was still alive, although the neurological disease had progressed with dysphagia and weakness affecting both upper and lower limb muscles (Table 1).

## DISCUSSION

ALS is a progressive neurodegenerative disorder that primarily affects motor neurons, leading to denervation, muscle atrophy, progressive paralysis, and a poor prognosis. The pathophysiology of ALS remains incompletely understood. Apart from cases linked to genetic mutations, the underlying mechanisms of motor neuron death remain unclear. Autoimmunity in ALS is suggested by the presence of circulating immune complexes, a higher frequency of specific histocompatibility subtypes and epidemiological data. Indeed, an association of ALS with prior diagnosis of certain autoimmune diseases such as type 1 diabetes, multiple sclerosis, Sjögren syndrome, systemic lupus erythematosus, or ulcerative colitis has been reported [5,6]. Neuroinflammation is a key feature of ALS, involving activated microglia and astrocytes in the central nervous system, as well as proinflammatory peripheral lymphocytes and macrophages. The role of TNFα in ALS is complex and not fully elucidated. TNFα levels are elevated in the serum and spinal cord of ALS animal models [7,8], and in humans, increased circulating TNFα and elevated levels in postmortem spinal cord tissue have been reported [9,10]. TNFα can exert both protective and deleterious effects, depending on receptor activation. Through nuclear factor kappa B signaling, TNFα promotes antiapoptotic and neurotrophic pathways, but it can also trigger proapoptotic effects. This duality is explained by the 2 receptors: p55/TNFR1, generally associated with cytotoxicity, and p75/TNFR2, associated with neurotrophic activity [11]. Cases of ALS under TNFi treatment have been reported mainly with monoclonal antibodies, with fewer cases under the fusion protein etanercept. This may reflect differences in the mechanism of action: monoclonal antibodies block TNFα binding to both receptors, whereas etanercept partially preserves p75/TNFR2 signaling. Despite promising animal data [12], clinical trials of TNFα blockade in ALS have not been conducted. Reports of ALS onset during TNFi therapy remain exceptional [[2], [3], [4]]. In 2012, a cluster of ALS cases was reported in the EudraVigilance database, the European system for adverse drug reaction reporting [13]. However, a large Swedish registry study found no increased rate of ALS in patients with RA treated with TNFi compared to the general population [14]. Analyses of the French Pharmacovigilance Database and the international Vigibase database suggested a potential but unconfirmed association between TNFi exposure and ALS onset [15]. However, an association between ALS and specific immunomodulators, including TNFi, has been reported by the Food and Drug Administration adverse event reporting system [16]. Overall, given the rarity of such cases, a causal relationship between TNFi and ALS development remains unproven and no definite conclusion can be made on this topic. Cases of ALS have not been reported on other biologics such as interleukin 6 inhibitors, rituximab, abatacept, or JAK inhibitor (JAKi). On the contrary, JAKi may decrease the process of neuroinflammation in ALS [17]. Nonetheless, an influence of TNFi on disease progression cannot be ruled out. Since many alternative treatments exist for inflammatory rheumatic diseases, continuing TNFi therapy in patients with ALS may increase the risk of infections, a frequent complication of ALS. Thus, initiating TNFi should be considered cautiously in patients with predisposing factors for ALS, such as a family history of the disease.
